# 1-(Phenyl­sulfon­yl)benzo[1,2:2′,3′]thieno[5′,4′-*b*]carbazole

**DOI:** 10.1107/S1600536810010044

**Published:** 2010-03-27

**Authors:** S. Thenmozhi, A. SubbiahPandi, V. Dhayalan, A. K. MohanaKrishnan

**Affiliations:** a Department of Physics, Presidency College (Autonomous), Chennai 600 005, India; b Department of Organic Chemistry, University of Madras, Guindy Campus, Chennai 600 025, India

## Abstract

In the title compound, C_24_H_15_NO_2_S_2_, the ring system composed of the five fused rings is almost planar (r.m.s. deviation for all non-H atoms = 0.056 Å). The dihedral angle between the fused ring system and the phenyl ring is 83.4 (9)°. The crystal packing is stabilized by C—H⋯π and π–π inter­actions between parallel ring systems [centroid–centroid distances = 3.526 (3), 3.877 (3) and 3.712 (3) Å].

## Related literature

For related structures, see: Murugavel *et al.* (2009 ▶); Chakkaravarthi *et al.* (2008 ▶); Ravishankar *et al.* (2005 ▶).
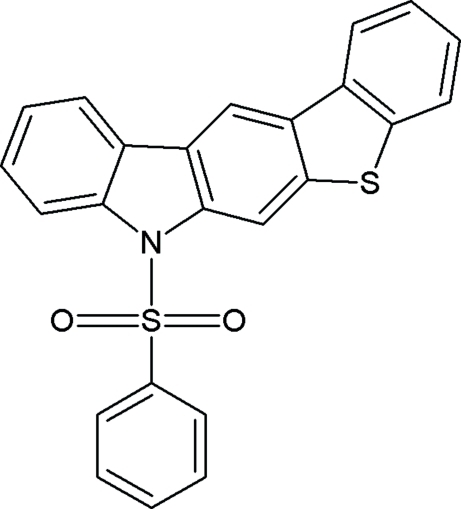


## Experimental

### 

#### Crystal data

C_24_H_15_NO_2_S_2_
*M* *_r_* = 413.49
Triclinic, 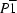

*a* = 7.463 (5) Å
*b* = 10.462 (5) Å
*c* = 12.335 (5) Å
α = 80.438 (5)°
β = 89.433 (5)°
γ = 81.876 (5)°
*V* = 940.1 (9) Å^3^
*Z* = 2
Mo *K*α radiation
μ = 0.31 mm^−1^
*T* = 293 K
0.25 × 0.22 × 0.19 mm

#### Data collection

Bruker APEXII CCD area-detector diffractometer
Absorption correction: multi-scan (*SADABS*; Sheldrick, 1996 ▶) *T*_min_ = 0.981, *T*_max_ = 0.985
24843 measured reflections
6428 independent reflections
4863 reflections with *I* > 2σ(*I*)
*R*_int_ = 0.024

#### Refinement

*R*[*F* ^2^ > 2σ(*F* ^2^)] = 0.057
*wR*(*F* ^2^) = 0.192
*S* = 1.01
6428 reflections
262 parameters
3 restraints
H-atom parameters constrained
Δρ_max_ = 0.98 e Å^−3^
Δρ_min_ = −0.62 e Å^−3^

### 

Data collection: *APEX2* (Bruker, 2004 ▶); cell refinement: *SAINT* (Bruker, 2004 ▶); data reduction: *SAINT*; program(s) used to solve structure: *SHELXS97* (Sheldrick, 2008 ▶); program(s) used to refine structure: *SHELXL97* (Sheldrick, 2008 ▶); molecular graphics: *ORTEP-3* (Farrugia, 1997 ▶); software used to prepare material for publication: *SHELXL97* and *PLATON* (Spek, 2009 ▶).

## Supplementary Material

Crystal structure: contains datablocks global, I. DOI: 10.1107/S1600536810010044/bt5204sup1.cif

Structure factors: contains datablocks I. DOI: 10.1107/S1600536810010044/bt5204Isup2.hkl

Additional supplementary materials:  crystallographic information; 3D view; checkCIF report

## Figures and Tables

**Table 1:** Hydrogen-bond geometry (Å, °) *Cg*6 is the centeroid of the C19–C24 ring.

*D*—H⋯*A*	*D*—H	H⋯*A*	*D*⋯*A*	*D*—H⋯*A*
C15—H15⋯*Cg*6^i^	0.93	2.78	3.685 (4)	166

Symmetry code: (i) 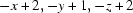.

**Fig. 1.**
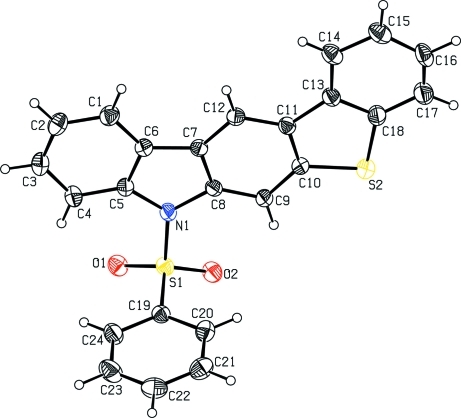
The structure of showing the atom-numbering scheme and intramolecular hydrogen bond. Displacement ellipsoids are drawn at the 30% probability level.

**Fig. 2.**
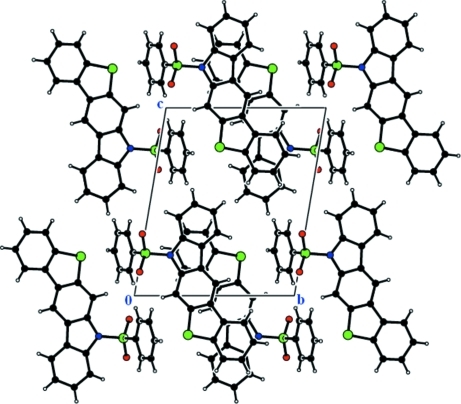
The molecular packing viewed down the a axis.

## supplementary crystallographic information

### Comment

Carbazole and its derivatives have become quite attractive compounds owing to their applications in pharmacy and molecular electronics.

In order to obtain detailed information on molecular conformations in the solid state, an X-ray study of the title compound was carried out.

The ring system composed of the five rings is almost planar (r.m.s. deviation for all non-H atoms 0.056Å). The crystal packing is stabilized by C–H..O and C–H···π (Table. 1) hydrogen bonds. In addition, there are π–π interactions between the extended ring system with a mean distance between the ring planes of 3.7Å.

### Experimental

To a solution of diethyl-2-((2-(bromomethyl)-1-(phenylsulfonyl)-1*H*-indol-3-yl) methylene)malonate (0.3 g 0.57 mmol) in dry 1,2-DCE (10 ml), InBr3 (0.02 g 0.06 mmol) and benzo[b]thiophene (0.09 g, 0.67 mmol) were added. The reaction mixture was stirred at room temperature for 4 h and then refluxed for 1 h under N2 atmosphere. It was then poured over ice-water (30 ml) containing 1 ml of Conc.HCl, extracted with chloroform (2 X 10 ml) and dried (Na_2_SO_4_). The removal of solvent followed by flash column chromatographic purification (silica gel, 230-420 mesh, n-hexane/ethyl acetate 99:1) afforded the product as a colorless solid. The product was recrystallization from CDCl_3_

### Refinement

All H atoms were fixed geometrically and allowed to ride on their parent C atoms, with C—H distances fixed in the range 0.93–0.97 Å with U_iso_(H) = 1.5U_eq_(C) for methyl H 1.2U_eq_(C) for other H atoms.

### Crystal data

**Table:**

C_24_H_15_NO_2_S_2_	*Z* = 2
*M**_r_* = 413.49	*F*(000) = 428
Triclinic, *P*1	*D*_x_ = 1.461 Mg m^−^^3^
Hall symbol: -P 1	Mo *K*α radiation, λ = 0.71073 Å
*a* = 7.463 (5) Å	Cell parameters from 6428 reflections
*b* = 10.462 (5) Å	θ = 1.7–32.2°
*c* = 12.335 (5) Å	µ = 0.31 mm^−^^1^
α = 80.438 (5)°	*T* = 293 K
β = 89.433 (5)°	Block, white crystalline
γ = 81.876 (5)°	0.25 × 0.22 × 0.19 mm
*V* = 940.1 (9) Å^3^	

### Data collection

**Table:**

Bruker APEXII CCD area-detector diffractometer	6428 independent reflections
Radiation source: fine-focus sealed tube	4863 reflections with *I* > 2σ(*I*)
graphite	*R*_int_ = 0.024
ω and φ scans	θ_max_ = 32.2°, θ_min_ = 1.7°
Absorption correction: multi-scan (*SADABS*; Sheldrick, 1996)	*h* = −11→11
*T*_min_ = 0.981, *T*_max_ = 0.985	*k* = −15→15
24843 measured reflections	*l* = −18→17

### Refinement

**Table:**

Refinement on *F*^2^	Primary atom site location: structure-invariant direct methods
Least-squares matrix: full	Secondary atom site location: difference Fourier map
*R*[*F*^2^ > 2σ(*F*^2^)] = 0.057	Hydrogen site location: inferred from neighbouring sites
*wR*(*F*^2^) = 0.192	H-atom parameters constrained
*S* = 1.01	*w* = 1/[σ^2^(*F*_o_^2^) + (0.1079*P*)^2^ + 0.4678*P*] where *P* = (*F*_o_^2^ + 2*F*_c_^2^)/3
6428 reflections	(Δ/σ)_max_ = 0.001
262 parameters	Δρ_max_ = 0.98 e Å^−^^3^
3 restraints	Δρ_min_ = −0.61 e Å^−^^3^

### Special details

**Table:**

Geometry. All esds (except the esd in the dihedral angle between two l.s. planes) are estimated using the full covariance matrix. The cell esds are taken into account individually in the estimation of esds in distances, angles and torsion angles; correlations between esds in cell parameters are only used when they are defined by crystal symmetry. An approximate (isotropic) treatment of cell esds is used for estimating esds involving l.s. planes.
Refinement. Refinement of *F*^2^ against ALL reflections. The weighted *R*-factor wR and goodness of fit *S* are based on *F*^2^, conventional *R*-factors *R* are based on *F*, with *F* set to zero for negative *F*^2^. The threshold expression of *F*^2^ > σ(*F*^2^) is used only for calculating *R*-factors(gt) etc. and is not relevant to the choice of reflections for refinement. *R*-factors based on *F*^2^ are statistically about twice as large as those based on *F*, and *R*- factors based on ALL data will be even larger.

### Fractional atomic coordinates and isotropic or equivalent isotropic displacement parameters (Å^2^)

**Table:**

	*x*	*y*	*z*	*U*_iso_*/*U*_eq_	
C1	0.8004 (3)	0.5479 (2)	0.66419 (19)	0.0477 (5)	
H1	0.8537	0.4628	0.6910	0.057*	
C2	0.7893 (3)	0.5939 (3)	0.5527 (2)	0.0538 (5)	
H2	0.8355	0.5391	0.5038	0.065*	
C3	0.7106 (3)	0.7203 (3)	0.51279 (18)	0.0517 (5)	
H3	0.7050	0.7491	0.4372	0.062*	
C4	0.6398 (3)	0.8051 (2)	0.58229 (17)	0.0456 (4)	
H4	0.5878	0.8904	0.5550	0.055*	
C5	0.6495 (3)	0.75792 (19)	0.69390 (15)	0.0367 (4)	
C6	0.7303 (3)	0.63112 (18)	0.73581 (16)	0.0368 (4)	
C7	0.7156 (2)	0.61248 (17)	0.85389 (15)	0.0347 (3)	
C8	0.6261 (2)	0.72886 (17)	0.88294 (15)	0.0344 (3)	
C9	0.5880 (3)	0.74249 (19)	0.99044 (16)	0.0394 (4)	
H9	0.5291	0.8202	1.0089	0.047*	
C10	0.6430 (3)	0.63321 (19)	1.06946 (15)	0.0380 (4)	
C11	0.7341 (2)	0.51533 (18)	1.04347 (15)	0.0360 (4)	
C12	0.7710 (3)	0.50503 (19)	0.93443 (16)	0.0388 (4)	
H12	0.8314	0.4279	0.9158	0.047*	
C13	0.7743 (3)	0.4149 (2)	1.13958 (16)	0.0402 (4)	
C14	0.8661 (3)	0.2850 (2)	1.1441 (2)	0.0502 (5)	
H14	0.9123	0.2541	1.0815	0.060*	
C15	0.8825 (4)	0.2076 (2)	1.2471 (2)	0.0564 (5)	
H15	0.9393	0.1215	1.2554	0.068*	
C16	0.8114 (3)	0.2611 (3)	1.34032 (18)	0.0531 (5)	
H16	0.8280	0.2079	1.4088	0.064*	
C17	0.7254 (4)	0.3785 (3)	1.3365 (2)	0.0593 (6)	
H17	0.6768	0.4077	1.3992	0.071*	
C18	0.7093 (3)	0.4578 (2)	1.23556 (18)	0.0482 (5)	
C19	0.6988 (3)	1.05404 (18)	0.76966 (16)	0.0378 (4)	
C20	0.7881 (3)	1.0581 (2)	0.86625 (19)	0.0478 (5)	
H20	0.7459	1.0208	0.9336	0.057*	
C21	0.9420 (3)	1.1189 (3)	0.8608 (2)	0.0579 (6)	
H21	1.0036	1.1228	0.9250	0.070*	
C22	1.0040 (4)	1.1735 (3)	0.7611 (3)	0.0606 (6)	
H22	1.1082	1.2133	0.7581	0.073*	
C23	0.9128 (4)	1.1697 (3)	0.6658 (2)	0.0648 (7)	
H23	0.9549	1.2078	0.5987	0.078*	
C24	0.7592 (3)	1.1098 (2)	0.6689 (2)	0.0517 (5)	
H24	0.6974	1.1070	0.6044	0.062*	
N1	0.5814 (2)	0.81927 (16)	0.78385 (13)	0.0385 (3)	
O1	0.4119 (2)	1.01545 (15)	0.67159 (13)	0.0470 (3)	
O2	0.4131 (2)	0.99247 (15)	0.87383 (13)	0.0461 (3)	
S1	0.50484 (6)	0.97634 (4)	0.77433 (4)	0.03684 (13)	
S2	0.60075 (9)	0.62317 (6)	1.20851 (5)	0.05295 (17)	

### Atomic displacement parameters (Å^2^)

**Table:**

	*U*^11^	*U*^22^	*U*^33^	*U*^12^	*U*^13^	*U*^23^
C1	0.0510 (11)	0.0457 (11)	0.0460 (11)	0.0000 (9)	0.0036 (9)	−0.0127 (9)
C2	0.0555 (13)	0.0620 (14)	0.0468 (12)	−0.0036 (10)	0.0082 (10)	−0.0223 (10)
C3	0.0546 (12)	0.0650 (14)	0.0358 (10)	−0.0087 (10)	0.0050 (9)	−0.0093 (9)
C4	0.0495 (11)	0.0495 (11)	0.0351 (9)	−0.0042 (9)	−0.0001 (8)	−0.0014 (8)
C5	0.0378 (9)	0.0393 (9)	0.0331 (8)	−0.0059 (7)	0.0009 (7)	−0.0055 (7)
C6	0.0359 (8)	0.0373 (9)	0.0375 (9)	−0.0053 (7)	0.0006 (7)	−0.0064 (7)
C7	0.0342 (8)	0.0341 (8)	0.0356 (8)	−0.0046 (6)	0.0002 (6)	−0.0053 (6)
C8	0.0366 (8)	0.0312 (8)	0.0346 (8)	−0.0055 (6)	0.0005 (6)	−0.0026 (6)
C9	0.0485 (10)	0.0346 (8)	0.0348 (9)	−0.0041 (7)	0.0029 (7)	−0.0070 (7)
C10	0.0439 (10)	0.0366 (9)	0.0348 (8)	−0.0096 (7)	0.0007 (7)	−0.0067 (7)
C11	0.0358 (8)	0.0364 (8)	0.0351 (8)	−0.0065 (7)	−0.0028 (7)	−0.0027 (6)
C12	0.0414 (9)	0.0351 (8)	0.0389 (9)	−0.0019 (7)	0.0001 (7)	−0.0065 (7)
C13	0.0391 (9)	0.0427 (8)	0.0385 (9)	−0.0114 (7)	−0.0037 (7)	−0.0006 (7)
C14	0.0469 (11)	0.0470 (9)	0.0523 (12)	−0.0071 (8)	−0.0063 (9)	0.0053 (9)
C15	0.0573 (13)	0.0452 (11)	0.0607 (12)	−0.0046 (10)	−0.0072 (10)	0.0071 (9)
C16	0.0505 (12)	0.0673 (12)	0.0383 (9)	−0.0205 (9)	−0.0106 (8)	0.0116 (8)
C17	0.0689 (15)	0.0661 (12)	0.0443 (11)	−0.0252 (10)	−0.0056 (11)	−0.0001 (10)
C18	0.0519 (12)	0.0587 (13)	0.0379 (10)	−0.0244 (10)	−0.0010 (8)	−0.0053 (9)
C19	0.0377 (9)	0.0340 (8)	0.0402 (9)	−0.0014 (7)	0.0009 (7)	−0.0051 (7)
C20	0.0466 (11)	0.0518 (12)	0.0438 (11)	−0.0014 (9)	−0.0003 (8)	−0.0093 (9)
C21	0.0500 (13)	0.0619 (14)	0.0647 (15)	−0.0057 (10)	−0.0092 (11)	−0.0199 (12)
C22	0.0475 (12)	0.0555 (14)	0.0813 (18)	−0.0152 (10)	0.0011 (12)	−0.0116 (12)
C23	0.0622 (15)	0.0656 (16)	0.0658 (16)	−0.0245 (13)	0.0082 (12)	0.0045 (12)
C24	0.0561 (13)	0.0546 (12)	0.0434 (11)	−0.0166 (10)	0.0006 (9)	0.0021 (9)
N1	0.0475 (9)	0.0335 (7)	0.0325 (7)	−0.0013 (6)	0.0013 (6)	−0.0033 (6)
O1	0.0434 (8)	0.0467 (8)	0.0464 (8)	0.0002 (6)	−0.0085 (6)	0.0007 (6)
O2	0.0452 (8)	0.0437 (8)	0.0460 (8)	0.0023 (6)	0.0109 (6)	−0.0060 (6)
S1	0.0362 (2)	0.0344 (2)	0.0371 (2)	−0.00038 (16)	0.00092 (17)	−0.00168 (16)
S2	0.0709 (4)	0.0497 (3)	0.0405 (3)	−0.0133 (3)	0.0044 (2)	−0.0102 (2)

### Geometric parameters (Å, °)

**Table:**

C1—C2	1.379 (3)	C14—C15	1.386 (3)
C1—C6	1.389 (3)	C14—H14	0.9300
C1—H1	0.9300	C15—C16	1.426 (4)
C2—C3	1.381 (4)	C15—H15	0.9300
C2—H2	0.9300	C16—C17	1.297 (4)
C3—C4	1.383 (3)	C16—H16	0.9300
C3—H3	0.9300	C17—C18	1.372 (3)
C4—C5	1.382 (3)	C17—H17	0.9300
C4—H4	0.9300	C18—S2	1.783 (3)
C5—C6	1.394 (3)	C19—C20	1.381 (3)
C5—N1	1.427 (2)	C19—C24	1.382 (3)
C6—C7	1.442 (3)	C19—S1	1.753 (2)
C7—C12	1.388 (3)	C20—C21	1.385 (4)
C7—C8	1.403 (3)	C20—H20	0.9300
C8—C9	1.380 (3)	C21—C22	1.371 (4)
C8—N1	1.426 (2)	C21—H21	0.9300
C9—C10	1.389 (3)	C22—C23	1.373 (4)
C9—H9	0.9300	C22—H22	0.9300
C10—C11	1.406 (3)	C23—C24	1.380 (4)
C10—S2	1.729 (2)	C23—H23	0.9300
C11—C12	1.389 (3)	C24—H24	0.9300
C11—C13	1.450 (3)	N1—S1	1.6469 (18)
C12—H12	0.9300	O1—S1	1.4212 (16)
C13—C18	1.393 (3)	O2—S1	1.4223 (16)
C13—C14	1.425 (3)		
			
C2—C1—C6	118.7 (2)	C13—C14—H14	122.0
C2—C1—H1	120.6	C14—C15—C16	119.5 (2)
C6—C1—H1	120.6	C14—C15—H15	120.3
C1—C2—C3	120.7 (2)	C16—C15—H15	120.3
C1—C2—H2	119.6	C17—C16—C15	124.7 (2)
C3—C2—H2	119.6	C17—C16—H16	117.6
C2—C3—C4	121.7 (2)	C15—C16—H16	117.6
C2—C3—H3	119.1	C16—C17—C18	116.9 (3)
C4—C3—H3	119.1	C16—C17—H17	121.5
C5—C4—C3	117.2 (2)	C18—C17—H17	121.5
C5—C4—H4	121.4	C17—C18—C13	122.7 (3)
C3—C4—H4	121.4	C17—C18—S2	125.7 (2)
C4—C5—C6	121.90 (19)	C13—C18—S2	111.53 (16)
C4—C5—N1	129.92 (19)	C20—C19—C24	121.4 (2)
C6—C5—N1	108.16 (16)	C20—C19—S1	119.55 (16)
C1—C6—C5	119.68 (19)	C24—C19—S1	119.07 (17)
C1—C6—C7	132.31 (19)	C19—C20—C21	118.7 (2)
C5—C6—C7	107.97 (16)	C19—C20—H20	120.7
C12—C7—C8	120.30 (17)	C21—C20—H20	120.7
C12—C7—C6	131.54 (17)	C22—C21—C20	120.4 (2)
C8—C7—C6	108.15 (16)	C22—C21—H21	119.8
C9—C8—C7	122.62 (17)	C20—C21—H21	119.8
C9—C8—N1	129.72 (17)	C21—C22—C23	120.3 (2)
C7—C8—N1	107.63 (16)	C21—C22—H22	119.9
C8—C9—C10	116.03 (18)	C23—C22—H22	119.9
C8—C9—H9	122.0	C22—C23—C24	120.5 (3)
C10—C9—H9	122.0	C22—C23—H23	119.7
C9—C10—C11	122.92 (18)	C24—C23—H23	119.7
C9—C10—S2	124.73 (16)	C23—C24—C19	118.7 (2)
C11—C10—S2	112.31 (14)	C23—C24—H24	120.6
C12—C11—C10	119.59 (17)	C19—C24—H24	120.6
C12—C11—C13	127.69 (18)	C8—N1—C5	108.07 (15)
C10—C11—C13	112.69 (18)	C8—N1—S1	126.27 (13)
C7—C12—C11	118.53 (17)	C5—N1—S1	124.47 (13)
C7—C12—H12	120.7	O1—S1—O2	120.28 (10)
C11—C12—H12	120.7	O1—S1—N1	106.82 (9)
C18—C13—C14	120.1 (2)	O2—S1—N1	106.80 (9)
C18—C13—C11	112.01 (19)	O1—S1—C19	108.43 (10)
C14—C13—C11	127.9 (2)	O2—S1—C19	108.38 (10)
C15—C14—C13	116.0 (2)	N1—S1—C19	105.12 (10)
C15—C14—H14	122.0	C10—S2—C18	91.45 (10)
			
C6—C1—C2—C3	0.1 (4)	C15—C16—C17—C18	−2.9 (4)
C1—C2—C3—C4	−0.1 (4)	C16—C17—C18—C13	2.7 (4)
C2—C3—C4—C5	−0.6 (4)	C16—C17—C18—S2	−179.09 (18)
C3—C4—C5—C6	1.3 (3)	C14—C13—C18—C17	−1.7 (3)
C3—C4—C5—N1	−176.6 (2)	C11—C13—C18—C17	177.6 (2)
C2—C1—C6—C5	0.5 (3)	C14—C13—C18—S2	179.82 (16)
C2—C1—C6—C7	177.8 (2)	C11—C13—C18—S2	−0.8 (2)
C4—C5—C6—C1	−1.3 (3)	C24—C19—C20—C21	−0.4 (3)
N1—C5—C6—C1	177.01 (18)	S1—C19—C20—C21	179.44 (18)
C4—C5—C6—C7	−179.18 (18)	C19—C20—C21—C22	−0.2 (4)
N1—C5—C6—C7	−0.9 (2)	C20—C21—C22—C23	0.7 (4)
C1—C6—C7—C12	1.5 (4)	C21—C22—C23—C24	−0.7 (5)
C5—C6—C7—C12	179.1 (2)	C22—C23—C24—C19	0.1 (4)
C1—C6—C7—C8	−177.7 (2)	C20—C19—C24—C23	0.4 (4)
C5—C6—C7—C8	−0.2 (2)	S1—C19—C24—C23	−179.4 (2)
C12—C7—C8—C9	−0.4 (3)	C9—C8—N1—C5	−179.31 (19)
C6—C7—C8—C9	178.97 (17)	C7—C8—N1—C5	−1.7 (2)
C12—C7—C8—N1	−178.20 (17)	C9—C8—N1—S1	12.8 (3)
C6—C7—C8—N1	1.1 (2)	C7—C8—N1—S1	−169.59 (14)
C7—C8—C9—C10	−0.3 (3)	C4—C5—N1—C8	179.7 (2)
N1—C8—C9—C10	176.99 (18)	C6—C5—N1—C8	1.6 (2)
C8—C9—C10—C11	0.8 (3)	C4—C5—N1—S1	−12.1 (3)
C8—C9—C10—S2	−176.55 (14)	C6—C5—N1—S1	169.78 (14)
C9—C10—C11—C12	−0.6 (3)	C8—N1—S1—O1	−156.86 (17)
S2—C10—C11—C12	177.07 (15)	C5—N1—S1—O1	37.10 (18)
C9—C10—C11—C13	−178.95 (18)	C8—N1—S1—O2	−27.0 (2)
S2—C10—C11—C13	−1.3 (2)	C5—N1—S1—O2	167.01 (16)
C8—C7—C12—C11	0.6 (3)	C8—N1—S1—C19	88.06 (18)
C6—C7—C12—C11	−178.54 (19)	C5—N1—S1—C19	−77.97 (17)
C10—C11—C12—C7	−0.2 (3)	C20—C19—S1—O1	164.32 (16)
C13—C11—C12—C7	177.95 (18)	C24—C19—S1—O1	−15.8 (2)
C12—C11—C13—C18	−176.85 (19)	C20—C19—S1—O2	32.19 (19)
C10—C11—C13—C18	1.4 (2)	C24—C19—S1—O2	−147.95 (18)
C12—C11—C13—C14	2.4 (3)	C20—C19—S1—N1	−81.72 (18)
C10—C11—C13—C14	−179.34 (19)	C24—C19—S1—N1	98.14 (19)
C18—C13—C14—C15	0.8 (3)	C9—C10—S2—C18	178.30 (19)
C11—C13—C14—C15	−178.4 (2)	C11—C10—S2—C18	0.71 (15)
C13—C14—C15—C16	−1.0 (3)	C17—C18—S2—C10	−178.3 (2)
C14—C15—C16—C17	2.2 (4)	C13—C18—S2—C10	0.08 (16)

### Hydrogen-bond geometry (Å, °)

**Table:**

Cg6 is the centeroid of the C19–C24 ring.

**Table:**

*D*—H···*A*	*D*—H	H···*A*	*D*···*A*	*D*—H···*A*
C15—H15···Cg6^i^	0.93	2.78	3.685 (4)	166

Symmetry codes: (i) −*x*+2, −*y*+1, −*z*+2.
